# Disease course following High Disease Activity Status revealed patterns in SLE

**DOI:** 10.1186/s13075-021-02572-1

**Published:** 2021-07-14

**Authors:** Alberta Hoi, Rachel Koelmeyer, Julie Bonin, Ying Sun, Amy Kao, Oliver Gunther, Hieu T. Nim, Eric Morand

**Affiliations:** 1 Centre for Inflammatory Diseases, School of Clinical Sciences, Monash University, Level 5, Block E, Monash Medical Centre, 246 Clayton Road, Clayton, VIC 3168 Australia; 2 Department of Rheumatology, Monash Health, Clayton, VIC 3168 Australia; 3 Merck Healthcare KGaA, Frankfurter Strasse 250, 64293 Darmstadt, Germany; 4 EMD Serono, EMD Serono Research & Development Institute, Inc, a business of Merck KGaA, Darmstadt, Germany; 5 Faculty of Information Technology, Monash University, Clayton, VIC 3168 Australia

## Abstract

**Background:**

We sought to examine the disease course of High Disease Activity Status (HDAS) patients and their different disease patterns in a real-world longitudinal cohort. Disease resolution till Lupus Low Disease Activity State (LLDAS) has been a general treatment goal, but there is limited information on this subset of patients who achieve this.

**Methods:**

All consenting patients of the Monash Lupus Cohort who had at least 12 months of observation were included. HDAS was defined as SLEDAI-2K ≥ 10 ever, and HDAS episode as the period from the first HDAS clinic visit until attainment of LLDAS. We examined the associations of different HDAS patterns with the likelihood of damage accrual.

**Results:**

Of 342 SLE patients, 151 experienced HDAS at least once, accounting for 298 HDAS episodes. The majority of HDAS patients (76.2%) experienced Recurrent HDAS (> 1 HDAS visit), and a smaller subset (47.7%) had Persistent HDAS (consecutive HDAS visits for longer than 2 months). Recurrent or Persistent HDAS patients were younger at diagnosis and more likely to experience renal or serositis manifestations; persistent HDAS patients were also more likely to experience neurological manifestations. Baseline SLEDAI greater than 10 was associated with longer HDAS episodes. Recurrent and Persistent HDAS were both associated with an increased likelihood of damage accrual. The total duration of HDAS episode greater than 2 years and experiencing multiple HDAS episodes (≥4) was also associated with an increased likelihood of damage accrual (OR 1.80, 95% CI 1.08–2.97, *p* = 0.02, and OR 3.31, 95% CI 1.66–13.26, *p* = 0.01, respectively).

**Conclusion:**

HDAS episodes have a highly variable course. Recurrent and Persistent HDAS, and longer duration of HDAS episodes, increased the risk of damage accrual. In addition to a major signifier of severity in SLE, its resolution to LLDAS can determine the subsequent outcome in SLE patients.

## Introduction

Systemic lupus erythematosus (SLE) is a chronic multisystem autoimmune disease with the potential to cause significant morbidity and mortality in affected patients. Clinical heterogeneity, in terms of breadth of organ involvement and disease activity over time, adds to the complexity of disease management and measurement. Tools with which to identify patients at high risk of adverse outcome are lacking. Recently, we described the concept of High Disease Activity Status (HDAS), defined as experiencing SLE Disease Activity Index-2000 (SLEDAI-2K) ≥ 10, as a disease severity and prognostic indicator [1], showing that attainment of HDAS at any time during the observation was associated with poorer long-term outcomes. In addition, in some clinical trials, this SLEDAI-2K cut-off has been associated with better discrimination of responders to targeted therapy [2–4], suggesting the possibility that such patients may be targeted for treatment escalation.

The natural history of SLE is relapsing and remitting, such that periods of higher disease activity are usually followed by lower disease activity, either spontaneously or in response to treatment escalation. However, the disease course following an occurrence of HDAS, and the resolution of such episodes, has not been described. In this study, we examine clinical associations of different HDAS patterns and their associated outcomes.

## Materials and methods

### Study design, setting, and participants

The Monash Lupus Clinic is a specialist outpatient clinic based at Monash Health in Melbourne, Australia. As a centre of the Australian Lupus Registry and Biobank (ALRB) [5], observational data are collected prospectively from patients with SLE. All participants provide written informed consent for their participation, and the study was approved by the Monash Health Human Research Ethics Committee. Data captured includes sociodemographic details, clinical laboratory and treatment information, and SLE-specific disease activity and damage assessments. SLE patients met either the American College of Rheumatology (ACR) or the Systemic Lupus International Collaborating Clinics (SLICC) SLE Classification Criteria prior to enrolment [6, 7]. The current study was limited to data from patients enrolled between April 2007 and July 2019 who had at least 2 clinic visits and at least 1 year of observation. As this was a retrospective analysis of prospectively collected data and no specific interventions were used, patients received standard of care.

### SLE-related clinical variables

Diagnostic assessments and autoantibody positivity were assessed at enrolment. Date of diagnosis refers to when the diagnosis of SLE was confirmed by a specialist. At each visit, SLE disease activity was measured using the SLEDAI-2K [8] and using the Physician Global Assessment and SLE Flare Index used in the Safety of Estrogens in Lupus Erythematosus National Assessment (SELENA) study [9]. Accrual of damage since the onset of SLE was measured annually using the SLICC-ACR Damage Index (SDI) [10].

### High Disease Activity Status (HDAS)

HDAS was defined as attainment of SLEDAI-2K ≥ 10 or greater [1]. We classified HDAS patients into Recurrent HDAS, which refers to those with at least two visits with SLEDAI-2K ≥10, or as Persistent HDAS, which is the subset of Recurrent HDAS patients who experienced at least one episode of SLEDAI-2K ≥ 10 for at least two consecutive visits over a period of at least 2 months. We also defined an HDAS episode as the period from the onset of HDAS until resolution, defined by attainment of Lupus Low Disease Activity State (LLDAS) [11, 12]. This construct allows us to study the disease course following patients’ presentation with HDAS. As disease activity can fluctuate depending on patient variability and treatment response, we defined the conclusion of the HDAS episode as the attainment of LLDAS.

### Statistical analysis

Descriptive statistics were used to describe the characteristics of included patients and the HDAS episodes. Bivariate tests (e.g., *χ*^2^ test, Fisher’s exact test, *t* test, and Kruskal-Wallis test) and multinomial logistic regression were used for group comparisons. Where applicable, unpaired heteroscedastic *t* tests were used assuming the variance of the groups compared was different. Logistic regression was used to assess the association of different HDAS groups, the number of HDAS episodes, and the duration of HDAS episodes with damage accrual. Other than pathology data, most other variables had a low level of missing data. Visits with missing SLEDAI-2K data (8.9% of visits) were excluded when calculating patterns of HDAS. A *p* = 0.05 was set as the threshold for statistical significance. Kaplan-Meier survival analysis was performed to examine the resolution of HDAS episodes in patients at different baseline SLEDAI-2K scores. A heatmap of HDAS episodes was generated using the *image.plot* function in the R (version 3.6.3) programming environment.

## Results

### Cohort characteristics

At the time of analysis, 406 SLE patients at the Monash Lupus Clinic were enrolled in the ALRB; 342 (84.2%) had at least 2 clinic visits and had been followed for at least 1 year and were therefore included in the analysis. These patients had a median (interquartile range, IQR) disease duration of 3.6 years (0.7–11.1 years) at enrolment and a mean (standard deviation, SD) observation time of 6.7 years (3.6 years). The median time-adjusted mean SLEDAI-2K (AMS) of the cohort was 3.6 (IQR 2.0–5.3).

### Patterns of HDAS occurrence

Almost half (151/342, 44.2%) of the participants experienced at least one HDAS visit (SLEDAI-2K ≥ 10); 10.5% only experienced HDAS at a single visit, and 33.6% had Recurrent HDAS (≥ 2 HDAS visits). Of the Recurrent HDAS group, 62.6% experienced at least one episode of Persistent HDAS.

Table 1 provides descriptive statistics of patient characteristics by HDAS category. One of the main determinants of whether patients experience Recurrent or Persistent HDAS was their duration of observation, as shown by the higher duration of observation. We also saw differences with respect to age of diagnosis, ethnicity, and serological activity. There were also higher frequencies of renal involvement, history of serositis, or neurological manifestations in Recurrent or Persistent HDAS patients (Table 1). To further explore the differences in patient characteristics, we conducted a multinomial logistic regression analysis (Table 2). The associations of these clinical features with Recurrent or Persistent HDAS were significantly higher. Recurrent or Persistent HDAS patients were also more likely to be treated with immunosuppressant (particularly mycophenolate, azathioprine, cyclophosphamide, and tacrolimus) and more likely to be on greater than 15 mg per day of prednisolone (Table 1).

**Table 1:** Patient characteristics by HDAS category (*n* = 342)

Patient characteristic	HDAS patient category				Evidence of difference in characteristic (*p* value)^c^
	Never experienced HDAS (*n* = 191)	One HDAS visit (*n* = 36)	Recurrent but not Persistent HDAS (*n* = 43)	Persistent HDAS (*n* = 72)	
**Female sex**	168 (88.0%)	30 (83.3%)	39 (90.7%)	59 (81.9%)	0.46
**Asian ethnicity**^a^	67 (35.8%)	16 (48.5%)	26 (61.9%)	33 (45.8%)	0.014
**Age at diagnosis (years)**, median (IQR)	32 (25, 45)	27 (19, 46.5)	26 (20, 37)	28.5 (21, 35)	0.012
**Disease duration at enrolment (years)**, median (IQR)	3 (0.6, 11.5)	4.6 (0.8, 11.2)	5.7 (2, 12.8)	4.05 (0.85, 10.3)	0.64
**Total patient observation time (years)**, median (IQR)	4.9 (3, 8.7)	5.6 (2.1, 8.1)	8.3 (4.7, 10.4)	8.8 (5.1, 12.1)	< 0.001
**Categorical observation time variable**					
< 5 years	98 (51.3%)	17 (47.2%)	12 (27.9%)	17 (23.6%)	< 0.001
5–10 years	62 (32.5%)	13 (36.1%)	17 (39.5%)	22 (30.6%)	
10+ years	31 (16.2%)	6 (16.7%)	14 (32.6%)	33 (45.8%)	
**Organ involvement**					
Skin	126 (66.0%)	18 (50.0%)	31 (72.1%)	45 (62.5%)	0.20
Arthritis	134 (70.2%)	23 (63.9%)	31 (72.1%)	44 (61.1%)	0.46
Haematological	92 (48.2%)	21 (58.3%)	27 (62.8%)	40 (55.6%)	0.26
Renal	43 (22.5%)	12 (33.3%)	24 (55.8%)	50 (69.4%)	< 0.001
Serositis	49 (25.7%)	11 (30.6%)	18 (41.9%)	38 (52.8%)	< 0.001
Neurological	16 (8.4%)	3 (8.3%)	7 (16.3%)	19 (26.4%)	0.001
**Serological profile**					
Anti_dsDNA	123 (65.1%)	30 (85.7%)	42 (97.7%)	70 (97.2%)	< 0.001
Anti_Sm	20 (11.0%)	8 (22.9%)	12 (28.6%)	17 (23.6%)	0.007
Anti_Ro	74 (40.7%)	17 (48.6%)	26 (61.9%)	38 (52.8%)	0.052
Anti-phospholipid-autoantibody-positive	94 (49.2%)	18 (50.0%)	25 (58.1%)	42 (58.3%)	0.49
Low complement at baseline^b^	89 (46.6%)	24 (66.7%)	29 (67.4%)	58 (80.6%)	< 0.001
Anti-dsDNA-positive and low complement at baseline	60 (31.4%)	20 (55.6%)	29 (67.4%)	57 (79.2%)	< 0.001
**Adjusted mean SLEDAI**, median (IQR)	2.2 (1.2, 3.5)	4.1 (3.55, 5.5)	5.2 (4.1, 6.2)	6.4 (5.0, 8.1)	< 0.001
**Mild/moderate flare rate**, median (IQR) per 100 person-years	0.3 (0, .6)	0.5 (0.3, 1.05)	0.9 (0.6, 1.2)	0.9 (0.6, 1.3)	< 0.001
**Severe flare rate**, median (IQR) per 100 person-years	0 (0, 0)	0.1 (0, 0.4)	0.2 (0.1, .4)	0.5 (0.2, 0.9)	< 0.001
**Damage accrual over observation period**	51 (26.7%)	13 (36.1%)	20 (46.5%)	48 (66.7%)	< 0.001
**Treatment received**					
Hydroxychloroquine	175 (91.6%)	32 (88.9%)	43 (100.0%)	69 (95.8%)	0.12
Other immunosuppressants^d^	119 (62.3%)	29 (80.6%)	41 (95.3%)	72 (100.0%)	< 0.001
Hydroxychloroquine/other immunosuppressants^d^	183 (95.8%)	35 (97.2%)	43 (100.0%)	72 (100.0%)	0.18
Mycophenolate	43 (22.5%)	15 (41.7%)	29 (67.4%)	56 (77.8%)	< 0.001
Azathioprine	53 (27.7%)	15 (41.7%)	24 (55.8%)	41 (56.9%)	< 0.001
Methotrexate	44 (23.0%)	9 (25.0%)	8 (18.6%)	19 (26.4%)	0.81
Cyclophosphamide	4 (2.1%)	0 (0.0%)	3 (7.0%)	17 (23.6%)	< 0.001
Tacrolimus	3 (1.6%)	0 (0.0%)	4 (9.3%)	4 (5.6%)	0.026
Leflunomide	11 (5.8%)	1 (2.8%)	1 (2.3%)	4 (5.6%)	0.73
Prednisolone (any dose)	128 (67.0%)	32 (88.9%)	41 (95.3%)	71 (98.6%)	< 0.001
Prednisolone > 15 mg per day	61 (31.9%)	20 (55.6%)	34 (79.1%)	64 (88.9%)	< 0.001

^a^89.1% (156) of non-Asians were of Caucasian ethnicity; other ethnicities reported included Aboriginal and/or Torres Strait Islander, African, Egyptian, Latin American, Maori, Middle Eastern, Pacific Islander, and Turkish ethnicity and persons of mixed ethnicity

^b^Includes positivity for anti-cardiolipin, anti-beta2GPI, or lupus anticoagulant

^c^Tests used to compare the HDAS categories included the Pearson’s chi-squared test, Fisher’s exact test, and the Kruskal-Wallis test

^d^Other immunosuppressants include mycophenolate, azathioprine, methotrexate, cyclophosphamide, tacrolimus, leflunomide, chloroquine, mercaptopurine, and sulfasalazine

**Table 2:** Clinical associations that predict HDAS patient categories

Patient characteristic	HDAS patient category^a^ *(relative risk, 95% confidence interval; p value)*		
	1 HDAS visit (*n* = 36)	Recurrent but not Persistent HDAS (*n* = 43)	Persistent HDAS (*n* = 72)
**Asian ethnicity**	1.69 (0.80–3.55; 0.170)	2.91 (1.46–5.81; 0.002)	1.52 (0.87–2.63; 0.140)
**Total patient observation (years)**	0.99 (0.89–1.09; 0.783)	1.13 (1.04–1.24; 0.006)	1.21 (1.12–1.30; < 0.001)
**Categorical observation time variable**			
< 5 years	1.0 (not applicable)	1.0 (not applicable)	1.0 (not applicable)
5–10 years	1.21 (0.55–2.66; 0.638)	2.24 (1.0–5.0; 0.050)	2.05 (1.0–4.15; 0.048)
10+ years	1.12 (0.40–3.08; 0.832)	3.69 (1.54–8.81; 0.003)	6.14 (3.01–12.49; < 0.001)
**Organ involvement by SLICC SLE Classification Criteria**			
Skin	0.52 (0.25–1.06; 0.071)	1.33 (0.64–2.77; 0.441)	0.86 (0.49–1.51; 0.599)
Arthritis	0.75 (0.36–1.59; 0.456)	1.10 (0.53–2.29; 0.801)	0.67 (0.38–1.18; 0.163)
Haematological	1.51 (0.73–3.10; 0.265)	1.82 (0.92–3.59; 0.086)	1.35 (0.78–2.32; 0.286)
Renal	1.72 (0.80–3.72; 0.168)	4.35 (2.18–8.68; < 0.001)	7.82 (4.27–14.33; < 0.001)
Serositis	1.28 (0.58–2.78; 0.541)	2.09 (1.05–4.15; 0.036)	3.24 (1.84–5.70; < 0.001)
Neurological	0.99 (0.27–3.60; 0.993)	2.13 (0.82–5.54; 0.123)	3.92 (1.88–8.16; < 0.001)
**Serological profile**			
Anti_dsDNA	3.22 (1.19–8.69; 0.021)	22.54 (3.03–167.46; 0.002)	18.78 (4.46–79.03; < 0.001)
Anti_Sm	2.40 (0.96–6.0; 0.061)	3.24 (1.43–7.32; 0.005)	2.50 (1.22–5.12; 0.012)
Anti_Ro	1.38 (0.67–2.85; 0.386)	2.37 (1.19–4.73; 0.014)	1.63 (0.94–2.82; 0.081)
Anti-phospholipid-autoantibody-positive	1.03 (0.51–2.10; 0.931)	1.43 (0.73–2.80; 0.292)	1.44 (0.84–2.50; 0.188)
Low complement at baseline	2.29 (1.08–4.85; 0.030)	2.37 (1.18–4.77; 0.015)	4.75 (2.48–9.09; < 0.001)
Anti-dsDNA-positive and low complement at baseline	2.73 (1.32–5.63; 0.007)	4.52 (2.23–9.17; < 0.001)	8.30 (4.35–15.82; < 0.001)

^a^Reference category: patients who never experienced HDA (*n* = 191)

### Characteristics of HDAS episodes

**Fig. 1:**
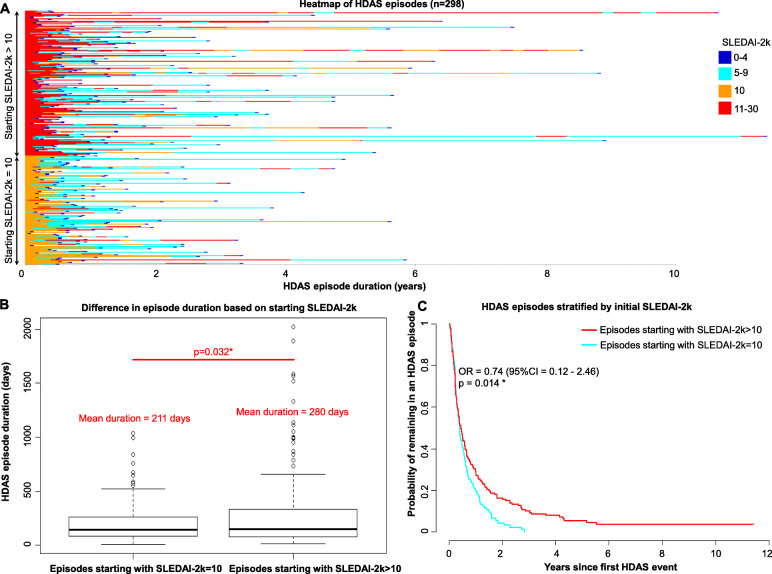
**A** Heatmap of HDAS episode duration and SLEDAI-2K over time. HDAS episodes (*n* = 298) are shown as rows, ordered by baseline SLEDAI-2K values. **B** Boxplot comparing the duration of HDAS episodes with baseline SLEDAI-2K > 10 versus baseline SLEDIA-2K-10. **C** Effect of baseline SLEDAI-2K on HDAS episode as shown by Kaplan-Meier survival analysis of recovery time of HDAS episodes between those with baseline SLEDAI-2K > 10 and SLEDAI-2K = 10

There were 298 HDAS episodes, defined as the period of observation between the first detection of HDAS until attainment of LLDAS, in 151 patients, observed over a total of 5193 visits. The duration of HDAS episodes was highly variable, with a median (IQR) of 147 days (84–294 days) (Fig. 1A). Disease activity at the commencement of an HDAS episode influenced episode duration. Compared to HDAS episodes where baseline SLEDAI-2K = 10, HDAS episodes with higher baseline scores (SLEDAI-2K > 10) were significantly longer (mean ± SD; 211 ± 200 vs 280 ± 354 days, *p* = 0.032) (Fig. 1B). The median time to resolution (LLDAS) was 5.5 months vs 5.1 months (OR 0.74, 95% CI 0.12 to 2.46, *p* = 0.014) (Fig. 1C), and the likelihood of HDA episodes lasting > 2 years was also significantly higher, with an odds ratio of 3.17 (95% CI = 1.04–9.6, *p* = 0.04).

### Different patterns of HDAS and damage accrual

Compared to non-HDAS patients, the risk of damage was markedly increased amongst patients with Recurrent or Persistent HDAS. When adjusted for observation time, Persistent HDAS patients had a significantly increased likelihood of damage accrual (OR 3.7, CI 2.0–7.1), *p* < 0.001 (Table 3). The duration of an HDAS episode was also strongly associated with damage accrual (Table 4). This was observed regardless of whether we calculated cumulative exposure of HDAS episodes for patients or individual HDAS episodes (Table 4). For HDAS episodes that lasted over 2 years, the adjusted odds ratio for damage accrual was 1.80 (95% CI 1.08–2.97, *p* = 0.02) (Table 4). We also examined the effects of multiple HDAS episodes on damage accrual and found that only experiencing 4 or more HDAS episodes was associated with increased damage accrual when adjusting for observation time (OR 3.31, 95% CI 1.66 to 13.26, *p* = 0.001) (Table 5).

**Table 3:** Risk of damage accrual by HDAS patient category

HDAS patient category	Odds of accruing damage			
	Unadjusted		Adjusted for observation time^a^	
	OR (95% CI)	*p* value	OR (95% CI)	*p* value
Never experienced HDAS	1	Not applicable	1	Not applicable
One HDAS visit only	1.6 (0.7–3.3)	0.252	1.6 (0.7–3.6)	0.267
All Recurrent HDAS	3.97 (2.43–6.49)	< 0.001	2.74 (1.60–4.69)	< 0.001
Recurrent but not Persistent HDAS	2.4 (1.2–4.7)	0.012	1.7 (0.8–3.6)	0.146
At least one episode of Persistent HDAS	5.5 (3.1–9.9)	< 0.001	3.7 (2.0–7.1)	< 0.001

^a^Adjusted for observation time modelled as a categorical variable (< 5 years, 5–< 10 years, and 10+ years)

**Table 4:** Risk of damage accrual by HDAS episode duration

HDAS episode duration	Odds of accruing damage			
	Unadjusted		Adjusted for observation time^a^	
	OR (95% CI)	*p* value	OR (95% CI)	*p* value
**Never experienced HDAS**	1	Not applicable	1	Not applicable
**Cumulative HDAS duration**				
≥1 day and < 1 year	1.64 (0.90–2.97)	0.12	1.48 (0.49–4.08)	0.46
≥1 year and < 2 years	1.70 (0.76–3.83)	0.19	1.27 (0.60–2.39)	0.49
2 years or more	5.55 (2.88–10.71)	< 0.001*	1.80 (1.08–2.97)	0.02*
**Mean duration of HDAS episode**				
≥1 day and < 1 year	2.31 (1.41–3.81)	< 0.001*	2.12 (0.88–5.00)	0.09
≥1 year and < 2 years	1.81 (0.71–4.62)	0.30	0.93 (0.27–2.31)	0.88
2 years or more	6.37 (2.30–17.66)	< 0.001*	1.60 (0.91–2.67)	0.08

*OR* odds ratio, *CI* confidence interval

*Statistically significant at *p* < 0.05

^a^Adjusted for observation time modelled as a categorical variable (< 5 years, 5–< 10 years, and 10+ years)

**Table 5:** Risk of damage accrual by number of HDAS episodes

Number of HDAS episodes	Odds of accruing damage			
	Unadjusted		Adjusted for observation time^a^	
	OR (95% CI)	*p* value	OR (95% CI)	*p* value
0	1	Not applicable	1	Not applicable
1	1.35 (0.72–2.51)	0.41	1.74 (0.63–4.5)	0.27
2	2.27 (1.13–4.55)	0.02*	1.09 (0.48–2.15)	0.83
3	2.23 (0.92–5.41)	0.08	1.44 (0.74–2.58)	0.23
4 or more	20.29 (5.80–70.98)	<0.001*	3.31 (1.66–13.26)	0.01*

*OR* odds ratio, *CI* confidence interval

*Statistically significant at *p* < 0.05

^a^Adjusted for observation time modelled as a categorical variable (<5 years, 5–<10 years, and 10+ years)

## Discussion

High Disease Activity Status (HDAS) has recently been confirmed as an important prognostic indicator for severe disease in SLE [1]. Studies of HDAS patients in real-world cohorts can shed light on how disease behaves in this group of patients which are typically the target population of clinical trials. In this study, we report on a number of disease patterns following an initial HDAS visit and their association with damage accrual. We observed that the majority of HDAS patients experience Recurrent HDAS rather than single events, a finding particularly evident with longer periods of observation suggesting that shorter studies may fail to detect this pattern. Recurrent or Persistent HDAS patients have the hallmark of a more severe phenotype, with younger at disease onset, more serological activity, and more serious organ involvement including renal disease, serositis, and neurological features.

We explored the relationship between different patterns of HDAS and damage accrual. Not surprisingly, Recurrent and Persistent HDAS were associated with increased risk of damage accrual even after adjusting for observation time. Our data also suggest that the total cumulative period of HDAS greater than 2 years, rather than the number of relapses, had a greater effect on damage accrual.

The attainment of LLDAS is an important treatment goal for people affected with SLE, as it has been associated with protection from flares and damage accrual [13]. In this study, we analysed the characteristics of HDAS episodes which were defined by the first attainment of LLDAS after patients experienced HDAS. Our study sheds light on the heterogeneity of the duration of these HDAS episodes. Longer duration of HDAS episodes and multiple recurrent HDAS episodes were strong predictors for damage accrual. Many factors can determine the duration of an HDAS episode, but one important consideration was the baseline disease activity. Higher SLEDAI-2K at the onset of HDAS was associated with longer HDAS episodes, which in turn was associated with an increased likelihood of damage accrual.

Monitoring the duration of an HDAS episode may be clinically relevant for planning treatment, as the relationship between the duration of HDAS episodes and damage appears to uphold whether patients experienced single or multiple HDAS episodes, implying that strategies aimed at attaining LLDAS more quickly after HDAS could limit the damage. Damage accrual was also significantly associated with patients who experienced multiple HDAS episodes (4 or greater), perhaps suggesting that this was a very select group with poor prognosis.

The implication of our findings is that early intervention to control disease activity upon the onset of HDAS is paramount. While a major challenge in SLE management is in the prevention of relapse, if disease can be rapidly brought into control, the risk of damage accrual can be minimised by curtailing the duration of the HDAS episode. The effect of multiple HDAS episodes on disease outcomes highlights the potential importance of maintenance therapy, particularly in the subset of patients who are prone to relapse or who have had a prolonged HDAS episode. Other studies report on baseline patient and disease characteristics as predictors of relapse which can guide clinician decision-making [14, 15].

A closer examination of HDAS episodes might also help us design better clinical trials. In our cohort, the median duration of HDAS episodes was about 5 months, suggesting any interventional trial starting at HDAS and using LLDAS as an efficacy endpoint should take this into account [3, 16, 17]. An important observation in this context is the longer time to resolution in patients whose HDAS episode began with a SLEDAI-2K > 10. Currently, most SLE clinical trials are designed with endpoints at 24 or 48 weeks [18]. If LLDAS is utilised as a primary endpoint, a shorter time to endpoint may allow greater differentiation between an active treatment arm and placebo when superimposed on the standard of care, particularly if we take into account the baseline SLEDAI-2K.

This study has some limitations. Some patients in our cohort entered into an HDAS episode but failed to achieve LLDAS during the observation period, for reasons such as shorter follow-up period or persistent disease activity. We have chosen not to include this smaller subset of patients in the current analysis as there may be many reasons for the inability to achieve LLDAS. Clinical manifestations at the time of HDAS varied considerably amongst patients, precluding analysis of associations of particular organ manifestations amongst HDAS patients with outcome. Although data were collected prospectively, this analysis is retrospective, and in addition, this is a single-centre study, albeit in a multi-ethnic cohort under universal health care. Confirmatory studies in independent cohorts are required, and we hope that the current findings will stimulate this.

## Conclusion

This study adds to existing evidence that HDAS is a poor prognostic indicator for SLE patients. In addition to previous findings that ever-attaining high disease activity defined as SLEDAI-2K ≥ 10, this study has demonstrated that Recurrent or Persistent HDAS, increased duration of HDAS episodes, and multiple repeated HDAS episodes were also associated with higher damage accrual. Future studies that examine for factors that predict the rapidity of HDAS resolution and maintenance of LLDAS will be useful.

## Data Availability

Ms Koelmeyer and Dr Nim had full access to all of the data in the study and take responsibility for the integrity of the data and the accuracy of the data analysis.

## Abbreviations

ACR: American College of Rheumatology
ALRB: Australian Lupus Registry and Biobank
AMS: Time-adjusted mean SLEDAI-2K
CI: Confidence interval
HDAS: High Disease Activity Status
IQR: Interquartile range
LLDAS: Lupus Low Disease Activity State
SD: Standard deviation
SDI: Systemic lupus erythematosus Damage Index
SELENA: Safety of Estrogens in Lupus Erythematosus National Assessment Study
SLE: Systemic lupus erythematosus
SLEDAI-2K: Systemic Lupus Erythematosus Disease Activity Index-2000
SLICC: Systemic lupus International Collaborating Clinics
